# Human Endogenous Retrovirus-K and TDP-43 Expression Bridges ALS and HIV Neuropathology

**DOI:** 10.3389/fmicb.2017.01986

**Published:** 2017-10-11

**Authors:** Renée N. Douville, Avindra Nath

**Affiliations:** ^1^Department of Biology, University of Winnipeg, Winnipeg, MB, Canada; ^2^Department of Immunology, University of Manitoba, Winnipeg, MB, Canada; ^3^Section of Infections of the Nervous System, National Institute of Neurological Diseases and Stroke, National Institutes of Health, Bethesda, MD, United States

**Keywords:** human immunodeficiency virus (HIV), human endogenous retrovirus-K (HERV-K), TDP-43, NeuroAIDS, amyotrophic lateral sclerosis (ALS)

## Abstract

Despite the repetitive association of endogenous retroviruses in human disease, the mechanisms behind their pathological contributions remain to be resolved. Here we discuss how neuronal human endogenous retrovirus-K (HERV-K) expression in human immunodeficiency virus (HIV)-infected individuals is a distinct pathological aspect of HIV-associated neurological conditions, such as HIV encephalitis and HIV-associated neurocognitive disorders. Enhanced neuronal HERV-K levels were observed in the majority of HIV-infected individuals, and to a higher degree in brain tissue marked by HIV replication. Moreover, we highlight an important neuropathological overlap between amyotrophic lateral sclerosis and HIV encephalitis, that being the formation of neurotoxic TDP-43 deposits in neurons. Herein, we argue for enhanced transdisciplinary research in the field of ERV biology, using an example of how HERV-K expression has novel mechanistic and therapeutic implications for HIV neuropathology.

## Introduction

Originating from ancient retroviruses that overcame host defense mechanisms and permanently integrated into the DNA of our hominid ancestors, endogenous retroviruses (ERVs) occupy over 8% of the human genome. The process of retroviral endogenation has resulted in at least 31 independently acquired ERV genera in the human genome (Belshaw et al., 2005; Blikstad et al., 2008; Subramanian et al., 2011). Despite roles in homeostasis (Brattas et al., 2017; Meyer et al., 2017), ERVs are increasingly being recognized as integral players in the pathogenesis of many human diseases [reviewed in Ruprecht et al. (2008), Balada et al. (2009), Manghera et al. (2014), Christensen (2016)]. Furthermore, concrete examples in other species indicate how ERVs may drive pathogenic mechanisms such as cellular transformation and immune dysregulation [reviewed in Kassiotis (2014), Mager and Stoye (2015)]. This is illustrated by mice deficient in select TLRs (-3, -7, -9), which exhibit endogenously derived MuLV viremia and development of acute T cell lymphoblastic leukemia (Yu et al., 2012). It also highlights the fact that both innate and adaptive immune responses are essential for control of ERV expression (Stetson et al., 2008; Hurst and Magiorkinis, 2015); conversely, ERVs also regulate immunity genes (Hurst and Magiorkinis, 2015; Chuong et al., 2016). Neurological effects caused by ERVs are also documented. Neurovirulent strains of MuLV such as Cas-Br-E cause infection of microglial cells with neurodegeneration in the brain and spinal cord which resembles amyotrophic lateral sclerosis (ALS) and is mediated by the envelope protein of the virus (Lynch and Sharpe, 2000; Jolicoeur et al., 2003).

Ongoing clinical trials seek to determine the value of ERVs as biomarkers and therapeutic targets (clinicaltrials.gov NCT02437110 and NCT02782858) (Alfahad and Nath, 2013). Breakthroughs in one specialty are likely to translate into other disease contexts, due to the overlap of ERV expression patterns in multiple conditions (Brutting et al., 2017). Herein, we argue for enhanced transdisciplinary research in the field of ERV biology, using an example of how endogenous retrovirus-K (HERV-K) expression has novel implications for human immunodeficiency virus (HIV) neuropathology.

## HIV Infection: More Than One Retrovirus At Play?

Even with antiretroviral therapy, the clinical treatment of HIV infection is often complicated by neurocognitive disorders (Robertson et al., 2007). The occurrence of mild neurocognitive deficits, such as those impacting daily living activities, have notably increased among HIV^+^ populations – despite a decline in the more severe forms of HIV-associated neurocognitive disorders (HAND) with the advent of antiretroviral therapy (McArthur et al., 2010). Even worse, there is currently no targeted treatment to prevent the onset or progression of HAND. Clinical trials with a wide variety of neuroprotective drugs, anti-inflammatory agents, and antioxidants have failed or shown only minor effects. Risk factors for the progression to HAND include genetic, viral, and co-morbid factors, such as drug use and increasing age (Saylor et al., 2016).

Human immunodeficiency virus enters the central nervous system (CNS) soon after initial infection. Whether HIV drives neurodegeneration by slow and progressive pathological changes or sudden alterations caused by systemic immunosuppression is unclear. In the CNS, productive HIV infection is supported by cell types such as astrocytes, microglia, and macrophages which subsequently elicit chronic pro-inflammatory responses [reviewed in Kraft-Terry et al. (2010)]. Both HIV infection and inflammatory signals mediated by cytokines such as IL-6, IL-1β, TNFα, and IFNγ, can trigger the expression of ERVs (Serra et al., 2003; Contreras-Galindo et al., 2007b), and specifically HERV-K (Manghera et al., 2016b).

Human immunodeficiency virus infection can promote ERV expression in proliferating peripheral blood mononuclear cells (PBMC) (Contreras-Galindo et al., 2007b). Longitudinal analysis of PBMC from HIV-infected patients shows that increased HERV-K expression precedes spikes of HIV replication in select individuals (Contreras-Galindo et al., 2007a). Enhanced HERV-K levels were observed in patients treated sub-optimal therapeutic doses of antiretrovirals, or those who outright failed to respond to HAART therapy (Contreras-Galindo et al., 2006, 2007a). Further evidence for a dynamically intertwined relationship between HERV-K and HIV replication stems form the observation that HIV elite controllers have robust cellular and antibody responses against the HERV-K (HML-2) capsid protein (de Mulder et al., 2017).

Until recently, it was unclear if HERV-K re-activation stays limited to peripheral tissues or is a feature of HIV neuroinvasion. Independent groups have now shown that HERV-K proteins accumulate in cortical neurons of patients with HIV infection (Bhat et al., 2014). Specifically, here we sought to determine if HIV infection in the brain is associated with the up-regulation of HERV-K expression, and additional markers of neuronal dysfunction.

## An Exploration of Herv-K Expression In HIV^+^ Brain Tissue

**FIGURE 1:**
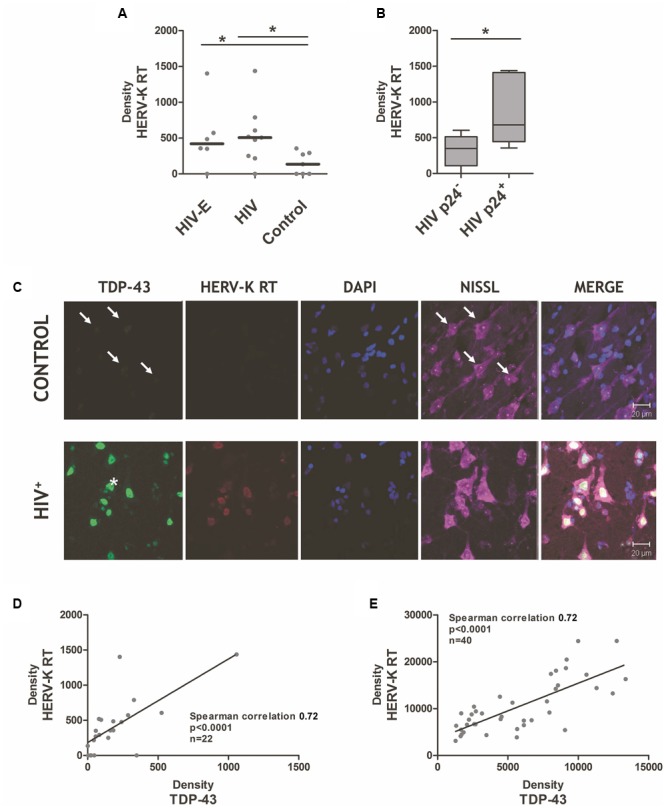
Endogenous retrovirus-K reverse transcriptase is induced in cortical tissue during human immunodeficiency virus (HIV) infection. **(A)** HIV-infected individuals, with HAND/HIV-encephalitis (HIV-E) or without HIV-E (HIV) expressed greater levels of endogenous retrovirus-K (HERV-K) reverse transcriptase protein in their cortical tissue, as compared to patients deceased with chronic systemic disease (control). Antibodies against the HERV-K reverse transcriptase protein (AbNova #H00002087-A01) and human TDP-43 (Protein Tech #10782-2-AP) were used for immunohistochemistry as previously described (Douville et al., 2011). **(B)** HIV replication in cortical tissue, as measured by HIV p24 protein immunostaining (mouse anti-HIV p24 Gag monoclonal, #24-4 NARRRP), is associated with significantly higher HERV-K reverse transcriptase expression. Mann–Whitney derived *t*-test, ^∗^*p* < 0.05. **(C)** Significant correlation of neuronal HERV-K reverse transcriptase and TDP-43 protein levels in HIV^+^ patients. Representative immunohistochemistry images of TDP-43 protein, endogenous retrovirus-K reverse transcriptase (HERV-K RT), nucleic as measured by DAPI staining, and neurons as measured by Nissl staining in the cortical brain tissue of HIV-infected patients (HIV^+^) versus patients with systemic disease (control). Arrows indicate weak TDP-43 positivity in control tissue neurons. ALS-derived neuron with a TDP-43 deposit outside the nuclear boundary is indicated with an asterisk. **(D,E)** A strong correlation between HERV-K RT and TDP-43 expression in brain tissues. ImageJ analysis was used to quantify the density of HERV-K RT and TDP-43 staining within individual tissue samples **(D)** and within individual neurons **(E)** of HIV-infected and controls cortical brain specimens. Spearman correlation used to assess association between HERV-K RT and TDP-43 expression patterns.

We have previously demonstrated enhanced HERV-K expression in cortical and spinal neurons of patients with ALS (Douville et al., 2011; Li et al., 2015). Therefore, we examined the extent of cortical HERV-K expression in HIV patients with encephalitis and/or HIV-associated neurocognitive disorder (HIV-E/HAND, *n* = 6,), HIV patients without encephalitis (HIV, *n* = 9), and individuals with chronic systemic illness (controls, *n* = 7), through detection and localization of the viral reverse transcriptase (RT) protein within autopsy tissues (**Table 1** and **Figure 1**).

**Table 1:** Cortical brain tissue specimens.

Case	Brain bank	Diagnosis	Tissue	Age (years)	Gender	PMI	CD4	Plasma viral load	CSF viral load
CB164	CNTC	HIV+	Parietal lobe	35	M	5	211	2827	78
CC128	CNTC	HIV+	Parietal lobe	47	M	12	306	4953	ND
CE132	CNTC	HIV+/HIV-E	Parietal lobe	39	M	7	26	ND	0
7101868276	TRANR	HIV+/HIV-E	Parietal cortex	41	M	7.6	37	>500000	>55695
7100616568	TRANR	HIV+/HIV-E	Parietal cortex	32	M	16.2	77	489796	3041500
7101847783	TRANR	HIV+	Parietal cortex	59	M	11.3	8	54439	735
7101997784	TRANR	HIV+	Parietal cortex	41	M	15.3	76	711	644
7100928374	TRANR	HIV+	Parietal cortex	38	F	10.8	3	104358	93
7100598287	TRANR	HIV+	Parietal cortex	50	M	13	121	<50	<10
1093	NNTC	HIV+/possible HAD	Parietal cortex	56	M	6	365	25669	ND
2005	NNTC	HIV+	Parietal cortex	44	F	3	66	>75000	249
2012	NNTC	HIV+/HIV-E	Parietal cortex	49	M	16	273	ND	<50
5007	NNTC	HIV+/possible HAD/HIV-E	Parietal cortex	37	F	3	91	>75000	ND
5008	NNTC	HIV+/possible HAD/HIV-E	Parietal cortex	53	M	7	128	8641	54061
6081	NNTC	HIV+/probable HAD	Parietal cortex	35	M	2	ND	ND	ND
3565	HBSFRC	Cardiomyopathy	Cortex	76	M	11			
27	RMMSC	Coronary artery disease	Cortex	69	M	6.5			
712	JHSMBB	Unknown	Prefrontal cortex	44	F	20			
23895	JHSMBB	Unknown	Occipital cortex	ND	ND	ND			
000	JHSMBB	Unknown	Motor cortex	ND	ND	ND			
33	RMMSC	Pulmonary disease	Cortex	69	M	4.5			
795	JHSMBB	Huntington’s disease	Sensory cortex	48	F	9			

*Tissue specimens were obtained from the California NeuroAIDS Tissue Consortium (CNTC), the Texas Repository for AIDS Neuropathogenesis Research (TRANR), the National NeuroAIDS Tissue Consortium (NNTC), the Human Brain and Spinal Fluid Resource Center (HBSFRC), the Rocky Mountain MS Center (RMMSC), and the Johns Hopkins School of Medicine Brain Bank (JHSMBB). Among confirmed cases with HIV infection (as indicated by CD4 count, plasma and cerebral spinal fluid viral loads), brain tissue samples were further classified as having HIV-encephalitis (HIV-E) based of neuropathological examination or HIV-associated dementia (HAD) based on clinical history. Post-mortem interval (PMI) is indicated in hours. No data (ND).*

In both HIV-infected groups, increased HERV-K RT protein was detectable (using AbNova #H00002087-A01 antibody), but surprisingly expressed at similar levels (**Figure 1A**). Based on the findings of Contreras-Galindo et al. (2007a), we expected HERV-K induction to occur immediately preceding or during HIV replication in the brain. HIV-E is a multifocal disease; therefore, varying levels of HIV replication occurs within the many regions of the brain. This leads to an intrinsic sampling bias when examining excised brain specimens, as select tissue samples may not be representative of the global pattern of HIV-associated pathology. Future studies would benefit from larger sample sizes and from sampling multiple brain regions per individual to improve the chances of identifying focal lesions. Considering these caveats, we evaluated the degree of HIV replication in each tissue specimen based on the presence or absence of HIV p24 positive cells as determined by immunohistochemistry, and stratified the tissues from HIV-infected individuals accordingly. HERV-K RT expression was significantly elevated in brain tissue with p24 reactivity (**Figure 1B**). This finding is consistent with the observation that productive HIV infection in PBMC triggers HERV-K expression (Contreras-Galindo et al., 2007b; Laderoute et al., 2007). Since HIV^+^ patients who fail to respond to HAART therapy exhibit elevated HERV-K levels in PBMC (Contreras-Galindo et al., 2007a), our results suggest that HERV-K expression in the brain may reflect inadequate drug penetration into the CNS or an unsuccessful response to antiretroviral treatment. Moreover, plasma HIV loads were associated with HERV-K RT measurements in cortical tissue (Spearman’s correlation *p* = 0.052), supportive of the idea that systemic HIV replication favors HERV-K re-activation in both the CNS and peripheral compartment. Productive HIV infection in neocortical tissues is characteristic of HIV-E/HAND (McArthur et al., 2010), and could also play a significant role in milder neurocognitive disorders. Moreover, robust HERV-K expression was seen in several HIV^+^ patients not clinically diagnosed with HIV-E/HAND. This points to pathological but modest HIV replication and/or HIV-associated neuroinflammation driving HERV-K activity within the brain, which precedes overt clinical signs and symptoms of neurocognitive impairment.

A notable finding is that the enhanced HERV-K expression was limited to neuronal cells in HIV^+^ specimens, yet HIV is not trophic for neurons. Local neuroinflammation is likely a key driver of HERV-K expression in the brain, and is supported in a study of ALS neuropathology (Manghera et al., 2016b). Alternatively, the enhanced neuronal expression of HERV-K may be directly mediated by viral proteins released from HIV-infected cells, such as HIV Tat (Gonzalez-Hernandez et al., 2012, 2014). HIV Tat protein can be released extracellularly from infected macrophages within the brain and then taken up by neurons [reviewed in Li et al. (2009)]. In depth analysis of the HERV-K promoter suggests that several transcription factors related to inflammation, hormone regulation, and tissue-specific signaling may also modulate the transcription of this ERV (Manghera and Douville, 2013).

However, a consequence of neuronal HERV-K upregulation may be a blockade on the progression of HIV infection in the brain. Experimental evidence suggests that HERV-K expression may be a limiting factor for HIV replication in neurons. *In vitro* modeling shows that HERV-K Gag and Env proteins can play an essential role as retroviral restriction factors, thus limiting HIV replication (Monde et al., 2017; Terry et al., 2017). Furin processing of HERV-K Env impacts HIV-1 production, as well as several key amino acid residues in the surface domain altering the degree of HIV production in HERV-K108 versus HERV-Kcon Env overexpressing 293T cells (Terry et al., 2017). Furthermore, it has been demonstrated that HERV-K Env (type 2) expression in neural cells is a protective mechanism against HIV replication in these cells, and that this may confer a degree of protection against a variety of insults (Bhat et al., 2014). Despite HERV-K activity impeding HIV replication in neurons, the brain remains negatively impacted by elevated HERV-K expression in neurons. Recent data show that the expression of HERV-K in mature neurons is toxic, with data also showing a neuropathological impact of HERV-K Env on mature neurons, as transgenic mice expressing HERV-K *env* are afflicted with severe neuronal damage and progressive motor dysfunction (Li et al., 2015). Another key point is that few studies have considered the potential roles of other HERV-K-encoded proteins on the viability and function of neurons. Clearly, we need a broader view of the impact of HERV-K in neurological disorders.

## The Search for An Herv-K-Associated Biomarker

Identification of HERV-K-associated biomarkers is a key step for both future research and clinical trials in a variety of HERV-K-associated diseases. Our previous study of patients with ALS found that the extent of HERV-K RT expression was strongly correlated with TAR DNA binding protein-43 (TDP-43) *in vivo* (Douville et al., 2011). Further, the HERV-K LTR has four binding sites for TDP-43 which have been shown to regulate its activation (Li et al., 2015). A common event in ALS is the aberrant deposit of ubiquitinated and hyper-phosphorylated TDP-43 in the cytoplasm and nucleus of neurons (Mackenzie et al., 2007; Buratti and Baralle, 2008). Further, formation of TDP-43 aggregates has been shown to alter HERV-K RT and polyprotein levels and cellular localization of these viral proteins (Manghera et al., 2016a). Measurement of TDP-43 protein expression (Protein Tech #10782-2-AP antibody) by immunohistochemistry (**Figure 1C**) or western blot analysis (data not shown) consistently show an increase in TDP-43 levels in HIV^+^ specimens, as compared to controls. Nuclear TDP-43 expression was enhanced on average six-fold in the cortical neurons of HIV patients, and was accompanied by enhanced TDP-43 phosphorylation (data not shown). Furthermore, co-expression of HERV-K RT and TDP-43 proteins occurred in the majority of neurons (**Figure 1C**). To examine this expression pattern quantitatively, the staining density measurements for HERV-K RT and TDP-43 were performed in tissues and co-labeled neurons. **Figures 1D,E** demonstrate that there is a significant positive correlation between TDP-43 expression and HERV-K RT expression in tissue (Spearman’s correlation 0.72, *p* < 0.0001, *n* = 22) and within individual neurons (Spearman’s correlation 0.72, *p* < 0.0001, *n* = 40). This data supports the idea that specific post-translational modifications of TDP-43 may alter its protein turnover rate resulting in modified nuclear expression, and the inception of cytoplasmic TDP-43 aggregates (**Figure 1C**, asterisk) (Mackenzie et al., 2007; Buratti and Baralle, 2008; Brady et al., 2011). This disruption in turn can impact HERV-K expression patterns (Manghera et al., 2016a).

As to why enhanced TDP-43 expression occurs in association with HERV-K, this phenomenon may reflect a similar transcriptional responsiveness to inflammatory signals. *In silico* analysis of the TDP-43 promoter reveals that like other interferon-stimulated genes, it harbors both IRF and κB binding sites and therefore may be transcriptionally up-regulated by pro-inflammatory responses. (Douville et al., 2011). HERV-K transcription and viral protein production are also strongly associated with the activity of the transcription factor complex of IRF1, and NF-κB p50 and p65 (Manghera et al., 2016b). Moreover, TDP-43 expression has been shown to be inducible during viral infection (Brasier et al., 2004). As TDP-43 was originally described as an inhibitor of HIV transcription (Ou et al., 1995), we postulated that it may act as a retroviral restriction factor, with a potential role in repressing both HIV and HERV-K provirus expression. Subsequent studies are not in agreement as to the potential of TDP-43 as a retroviral restriction factor, with some in support (Li et al., 2015; Krug et al., 2017), and others against (Nehls et al., 2014; Manghera et al., 2016a; Prudencio et al., 2017), perhaps reflecting cell-type or model-specific effects.

**Figure 1C** also highlights an important neuropathological overlap between ALS and HIV-E: the formation of nuclear and cytoplasmic TDP-43 deposits in neurons. The cleavage and subsequent aggregation of TDP-43 results in neurotoxicity (Yang et al., 2010; Che et al., 2011; Ratti and Buratti, 2016). There is a preponderance of TDP-43 pathology in over 90% of ALS cases (Irwin et al., 2015; Scotter et al., 2015), despite <1% of sporadic ALS cases having a clear genetic cause (Ling et al., 2013). Similarly, we observed that all HIV^+^ brain tissues exhibited heightened TDP-43 expression, with select neurons containing cytoplasmic TDP-43 accumulation (**Figure 1C**, asterisk). Truncated forms of TDP-43 called TDP-25 and TDP-35 are known to seed native TDP-43 aggregation (Huang et al., 2014; Xiao et al., 2015), thus altering its capacity to perform cellular functions (Che et al., 2011; Monahan et al., 2016). With sequestration of TDP-43, the cell’s ability to regulate RNA splicing is compromised (Scotter et al., 2015). Thus, HIV neuroinfection and ALS may share aberrant and altered protein deposition patterns, with pathogenic consequences.

The role of retroviruses (if any) in the cell-to-cell transmission of pathogenic TDP-43 moieties has yet to be elucidated (reviewed in Hanspal et al. (2017)]. This represents an attractive hypothesis considering that cortical neurons are known to secrete exosomes (Faure et al., 2006), and that exosomes have been shown to harbor retroviral cargo (Wurdinger et al., 2012; Vargas et al., 2014). Monitoring exosome composition could be an attractive source of clinical biomarkers for future studies on ERV-associated diseases.

## Future Directions

Despite repetitive association of ERVs in neurological diseases, such as ALS, multiple sclerosis, and schizophrenia, the mechanisms behind their pathological contributions remain to be resolved. Accumulating evidence suggests that HERV-K expression is a distinct pathological aspect of HIV-associated neurological disorders. Enhanced HERV-K RT expression in adult HIV^+^ individuals was restricted to neurons, and most elevated within brain tissue exhibiting HIV replication. This is a similar pattern of neuropathology to that seen in ALS, where cortical neurons – including motor neurons – express HERV-K viral proteins (RT and envelope proteins) (Douville et al., 2011; Bhat et al., 2014; Li et al., 2015; Manghera et al., 2016b). Considering this notable association with motor neuron disease, it is important to point out the prevalence of motor disturbances in HIV infection. Perinatally-acquired HIV infection is associated with neurodevelopmental disturbances, including neurocognitive, gross motor, and psychomotor deficits (Blanchette et al., 2001; McGrath et al., 2006). Elevated rates of motor dysfunction and developmental delay is widely reported in HIV+ infants and children across socio-economic strata, with reported rates reaching 66.7% (Ferguson and Jelsma, 2009; Govender et al., 2011; Le Doare et al., 2012). Even seemingly asymptomatic HIV^+^ children exhibit measurable motor deficits in standardized testing (Boivin et al., 1995). In adult HIV populations, motor symptoms were often found in conjunction with HAND, but in the post-HAART era it is less common to clinically observe deficits in motor skills and psychomotor speed (Heaton et al., 2011; Saylor et al., 2016). In both children and adults, early initiation of combination antiretroviral therapy (cART) has been shown to improve motor function (despite remaining subnormal), but later initiation of cART failed to yield as notable clinical improvement (Le Doare et al., 2012; Kore et al., 2015; Saylor et al., 2016). The potential role of HERV-K in mediating neurodevelopmental abnormalities in the HIV-infected children needs to be explored. Importantly, HERV-K plays a critical role in early embryogenesis (Grow et al., 2015), and hence its dysregulation by HIV could potentially alter neurodevelopment.

In rare cases, an ALS-like syndrome can occasionally be caused by retroviruses such as HIV and human T cell leukemia virus type-1 (HTLV-1) (Matsuzaki et al., 2000; Verma and Berger, 2006). Antiretroviral therapy can reverse the symptoms of this ALS-like syndrome in HIV-infected individuals (Moulignier et al., 2001; von Giesen et al., 2002), suggesting that HIV replication in the CNS can drive a pathology which symptomatically resembles ALS. Indeed, recent attempts treat HIV-associated motor neuron disease with antiretroviral therapy showed promise, with reversal of recent onset symptoms or a protracted course of the illness following treatment (Bowen et al., 2016). In these patients, the clinical improvement paralleled a decrease in HERV-K viral load in plasma (Bowen et al., 2016). Future research into HIV-associated motor deficits in pediatric and adult populations should consider the potential of HERV-K in driving motor impairment.

Our results support the concept that a failure to control HIV infection, either from a lack of response to HAART therapy, receiving a sub-optimal regimen or the inability of antiretroviral drugs to adequately penetrate the CNS, may be associated with enhanced HERV-K in the brain. The use of antiretroviral drugs to suppress HIV replication in the CNS may have an indirect (or potentially direct) neuroprotective effect by limiting HERV-K-mediated pathology. There is currently a dearth of knowledge pertaining to the identification and use of antiretrovirals customized for use against HERV-K (Tyagi et al., 2017). It remains to be seen how optimization of drug regimens more tailored to inhibition of HERV-K proteins may improve upon these clinical results.

Cognitive and psychomotor symptoms in HIV have been associated with structural changes in the brain following HIV infection, enhanced inflammation and immune activation, as well as metabolic disturbances (Saylor et al., 2016). By merging transdisciplinary expertise, our findings point to an overlapping pathological contribution of TDP-43 deregulation and HERV-K re-activation in both HAND and ALS. Exploration into the pathogenic effects of TDP-43 proteinopathy in ALS has vastly expanded potential therapeutic options for this neurological disease, including small molecule activators of autophagy, the ubiquitin-proteasome system, and chaperone proteins (Scotter et al., 2015). Indeed, therapeutics overcoming TDP-43-mediated pathology may also be clinically useful in retrovirus-associated neurological disease. An improved understanding of the role of TDP-43 in the pathogenesis of HERV-K and HIV infection may hold benefit for either ALS or HAND, in addition to other TDP-43-associated disorders such as Alzheimer’s disease (Davis et al., 2017) and prefrontal dementia (Lomen-Hoerth et al., 2003; Merrilees et al., 2010). Considering the seemingly disparate human conditions associated with ERV expression, more efforts into bridging disciplines is warranted, with the goal of elucidating shared molecular pathways and pathologies. Elevating the field of ERVs into the realm of mainstream biomedical research is worthwhile given its considerable clinical relevance. New outlooks on how ERV biology fits into our understanding of human disease may bring us closer to treating some of the most clinically difficult conditions, such as HIV-associated neurological disorders and ALS.

## Ethics Statement

De-identified autopsy brain samples were obtained from the National Neuro-AIDS Tissue Consortium (www.nntc.org). The use these tissues was determined to be IRB exempt.

## Author Contributions

RD and AN designed the study and wrote the manuscript. RD performed the experiments and analyzed the data. All authors read and approved the final manuscript.

## Conflict of Interest Statement

The authors declare that the research was conducted in the absence of any commercial or financial relationships that could be construed as a potential conflict of interest.
